# The County Infirmary, Leicester

**Published:** 1897-11-13

**Authors:** 


### The Institutional Workshop.

### THE COUNTY INFIRMARY, LEICESTER.

### ITS INFLUENCE AND PRESTIGE.

Amidst all the many manifestations of rejoicing which have exhibited themselves during the Diamond Jubilee Year, few, if any, are more remarkable or worthy of closer study than those connected with the Leicester Infirmary bazaar. The County Infirmary, though one of the oldest institutions in the country, which used before the days of county lunatic asylums to provide for those suffering not only from bodily but from mental ailments, still continues to attract to its work the interest and support of all classes of residents. People are proud of the County Infirmary, and the more ambitious amongst them aspire to a seat on the infirmary boards. Not only do the county aristocracy take these institutions under their wing, but in some instances, as at Leicester, the annual contributions from the working classes exceed the amount of annual subscriptions received from the upper and middle classes. Leicester has grown in population and increased in prosperity especially during the last thirty years, and is now one of the most vigorous of county towns. The infirmary bazaar, under the able guidance of Mrs. Edwin De Lisle (the president), was so well organised, so artistically arranged, and so wonderfully attractive as to constitute a notable feature of the year of commemoration. The Floral Hall, in which it was held, presented a charming scene, the stalls including an Indian palace with all its Eastern features, a picturesque South African house, models of Buckingham Palace, Windsor and Balmoral Castles, Osborne House, Sandringham, and St. James's Palace. Australasia, too, was represented by some homes of settlers placed in the midst of palm groves and tree ferns. A feature of the organisation was an arrangement by which the bazaar was opened on each of the four days by a lady or gentleman of influence, viz., the Duchess of Rutland, the Countess of Lonsdale, the Mayor of Leicester, and the Countess of Warwick respectively. We have not space to report the speeches as a whole, but it is due to the Countess of Warwick, who takes such infinite pains to secure the utmost advantage of any institution which is fortunate enough to enlist her sympathies, that we should report her speech on the occasion. This speech contains some good points, which most hospital committees and officers would do well to consider. It is not a little remarkable, too, that the receipts on the fourth day, when Lady Warwick opened the bazaar, viz., £519, appear from the local papers to have been the largest of all. The total for the four days is given as £2,228, which includes, we understand, a donation of £500 given by one gentleman to endow a cot. If this be the fact, it is an object-lesson to those who have to organise these functions, for it proves that success is largely dependent upon enlisting the aid of those who, as in the case of Lady Warwick, throw themselves heart and soul into the occasion, take infinite pains, and so secure a record.

The Countess of Warwick spoke as follows:—

#### County versus Cottage Hospitals.

She felt it a great honour, she said, and a great pleasure to be asked to come there that day to open that bazaar on, she believed, the fourth day, and to take part with them in helping on this good work which they had undertaken on behalf of the Leicester Infirmary. The last time she was in Leicester she stood on that platform to open a bazaar, which was also for a very good purpose, and it was a very great pleasure to her to feel that they were kind enough to invite her to come again and take part in those proceedings. The Leicester Infirmary held, she believed, a unique position among county hospitals. Its influence in the county of Leicester is so paramount that it is the only large county in England where the cottage hospital movement has not been warmly taken up. She found, on looking up the reports, that until 1891, although cottage hospitals had been in existence since 1859, Leicester contained no cottage hospital, but in that year one containing six beds was established at Hinckley, to which a district nurse was attached, who paid on an average upwards of 4,000 visits a year. There was also a small hospital at Mountsorrel, erected by the colliery proprietors for the use of their own men, which she must not omit to mention; but apart from these the Leicester Infirmary stood out as the great hospital for the county, and she was sure that was the reason that that bazaar was creating such a widespread interest.

#### Economy and Efficiency.

There seemed also another reason that the Leicester Infirmary occupied a unique position, and that was, that whereas hospitals existed chiefly for the working classes, during the last few years, while considerable discussion had arisen as to the share taken by the working classes in support of hospitals, the subscriptions of the working classes of Leicester already amounted to a noble sum, and this fact should encourage them to complete the work they had taken in hand, as it should also stimulate other classes to cheerfully provide the additional income which was required to bring their contributions up to the average at which they certainly ought to stand. With regard to the population, the Leicester Infirmary deservedly held a high place for the efficiency of its nursing, and the economical system upon which it had been conducted was proved by the fact that the average cost per occupied bed was about £52 per year, a sum which could not properly be reduced without risk to the efficiency and well-being of the institution. These facts reflected the highest credit on the committee and officers, to which the grateful recognition of the community was certainly due.

#### A Centre for Education.

The county infirmary must always occupy a position of influence, and should form a centre for the education and encouragement of the community in sound principles of hygiene and nursing and economical principles, upon the enforcement of which the highest success in the administration of an institution must mainly depend. For every hospital, every county infirmary, should be so managed as to secure that its bookshelves should contain all the standard literature on the subject, and especially such current literature and periodicals which dealt with the questions which it was the high privilege of the medical staff, the committee, and officers to thoroughly master and teach. She was sure that she need not ask if in the library attached to the Leicester Infirmary they could find a copy of "Hospitals and Asylums of the World," and a complete series of Burdett's "Hospitals and Charities," so that any governor or member of the public might follow the progress of the institution and compare its condition with other similar hospitals. This possibility of comparison was most important. Without adequate knowledge unintelligent criticism often arose, which tended to create a feeling of suspicion in the public mind, for which very frequently there was no sound reason, and which would never have arisen had the hospital contained, by the possession of such books as she had referred to, the means of using the actual facts for the guidance and information of the governors and the public generally. In these days of improved education, where all classes were coming to realise the importance of knowledge, it was the duty of the managers of every great hospital to assure themselves that they were in reality making progress upon the right lines, and to be in a position to enable everybody interested in their work to convince themselves that such was the case, by having facilities of access to the necessary books and publications where neutral and convincing evidence is to be found.

"The dreams of yesterday are the realities of to-day;
New occasions teach new duties,
Time makes ancient good uncouth,
They must upwards still and onward, who would keep abreast of truth."

